# Supplementary material for: Selective Excitation of Lanthanide Co-Dopants in Colloidal Lead-Free Halide Perovskite Nanocrystals as a Multilevel Anti-Counterfeiting Approach
Source: Nanomaterials (Basel). 2025 Dec 5;15(24):1838. doi: 10.3390/nano15241838 (PMC12735541; doi:10.3390/nano15241838)
Supplement: Supplementary file 1 [file nanomaterials-15-01838-s001.zip › nanomaterials-4015208 SI.pdf]

nanomaterials

Supplementary materials

Selective Excitation of Lanthanide Co-Dopants in Colloidal Lead-Free Halide Perovskite Nanocrystals as a Multilevel Anti-Counterfeiting Approach

Olexiy Balitskii *,† , Wilson Kagabo † and Pavle V. Radovanovic *

Department of Chemistry, University of Waterloo, 200 University Avenue W., Waterloo, ON N2L3G1, Canada
* Correspondence: olexiy.balitskii@uwaterloo.ca (O.B.); pavler@uwaterloo.ca (P.V.R.)
† These authors contributed equally to this work.

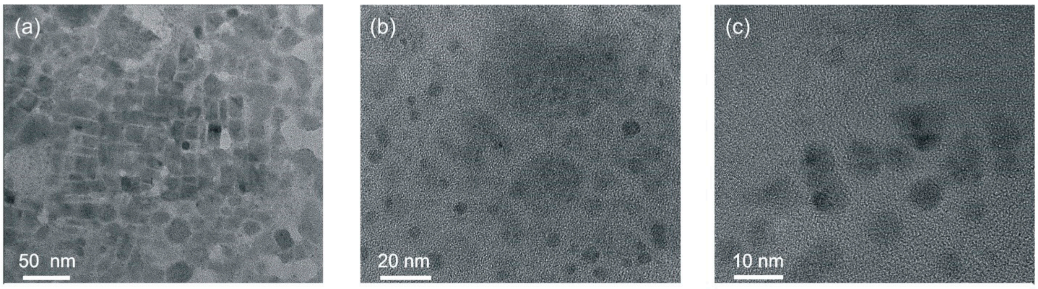

Figure S1. Overview TEM images of $Sb^{3+}/Dy^{3+}/Eu^{3+}$-co-doped CNYC (a), CNIC (b), and CNGC (c) DHP NCs with a nominal doping concentration of 5%/12.5%/12.5%.

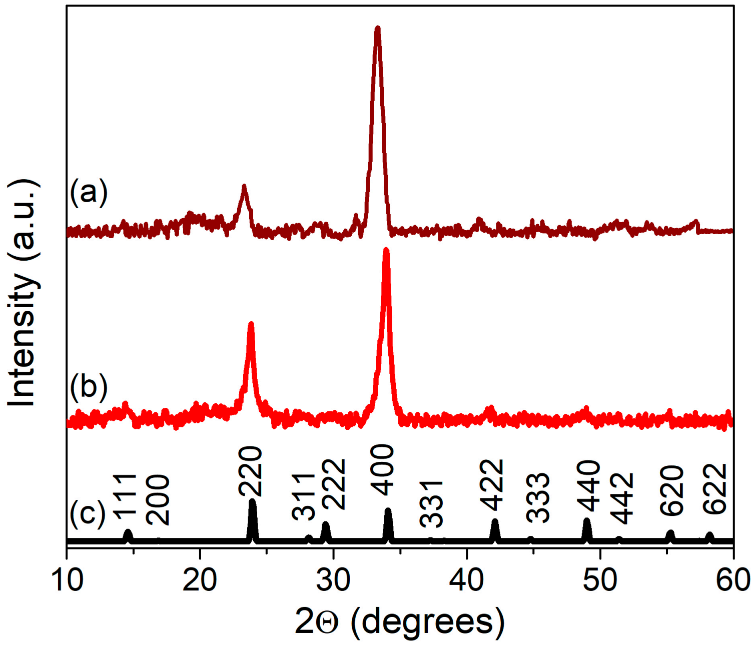

Figure S2. XRD patterns of $Sb^{3+}/Dy^{3+}/Eu^{3+}$-co-doped CNGC (a), CNIC (b) NCs with a nominal doping concentration of 5%/12.5%/12.5%, and ICSD132718 (c) for $Cs_2NaInCl_6$ DHP.

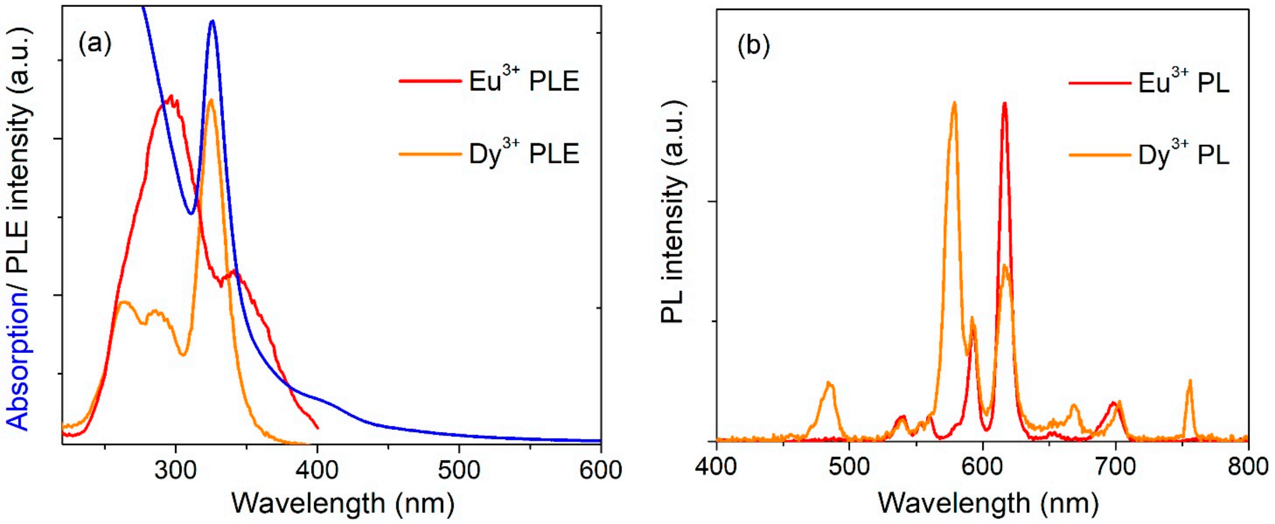

Figure S3. (a) Absorbance/PLE and (b) delayed PL spectra of Bi^{3+}/Dy^{3+}/Eu^{3+}-co-doped CNIC NCs with nominal doping concentrations of 5%/12.5%/12.5%. For delayed PL measurements, the NCs were excited at Bi^{3+} absorbance maximum (corresponding PLE recorded for $^4F_{9/2}$–$^6H_{13/2}$ Dy^{3+} line at 576 nm) and at 297 nm (corresponding PLE recorded for $^5D_0$–$^7F_2$ Eu^{3+} line at 617 nm).

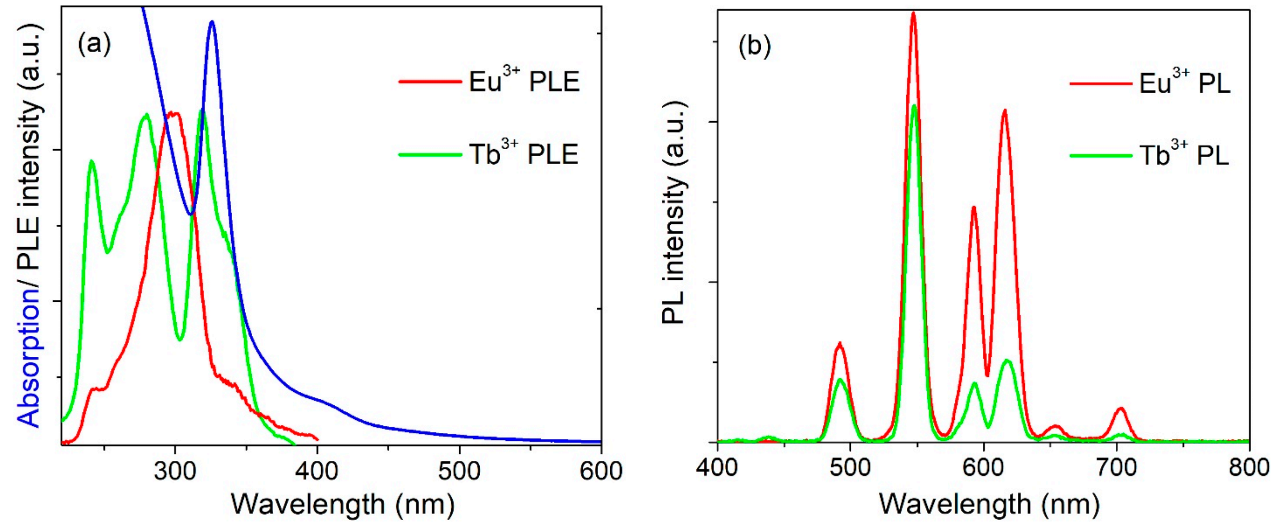

Figure S4. (a) Absorbance/PLE and (b) delayed PL spectra of Bi^{3+}/Tb^{3+}/Eu^{3+}-co-doped CNIC NCs with nominal doping concentrations of 5%/12.5%/12.5%. For delayed PL measurements, the NCs were excited at Bi^{3+} absorbance maximum (corresponding PLE recorded for $^5D_4$–$^7F_5$ Tb^{3+} line at 548 nm) and at 297 nm (corresponding PLE recorded for $^5D_0$–$^7F_2$ Eu^{3+} line at 617 nm).

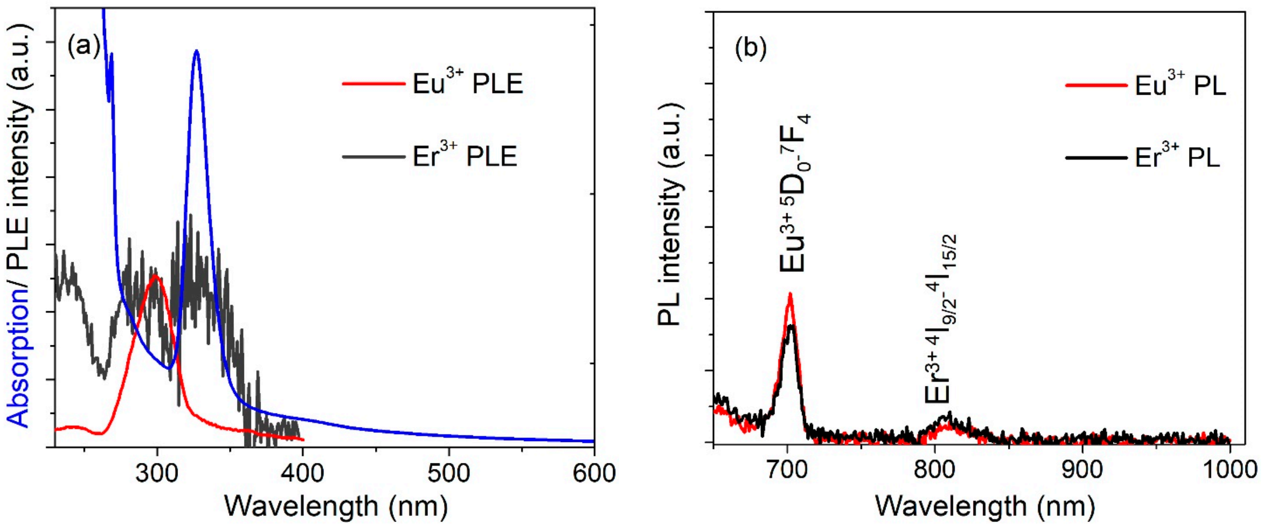

Figure S5. (a) Absorbance/PLE and (b) delayed PL spectra of $Bi^{3+}/Er^{3+}/Eu^{3+}$-co-doped CNIC NCs with nominal doping concentrations of 5%/12.5%/12.5%. For delayed PL measurements, the NCs were excited at Bi^{3+} absorbance maximum (corresponding PLE recorded for $^4I_{9/2}$–$^4I_{15/2}$ Er^{3+} line at 809 nm) and at 297 nm (corresponding PLE recorded for $^5D_0$–$^7F_4$ Eu^{3+} line at 703 nm).

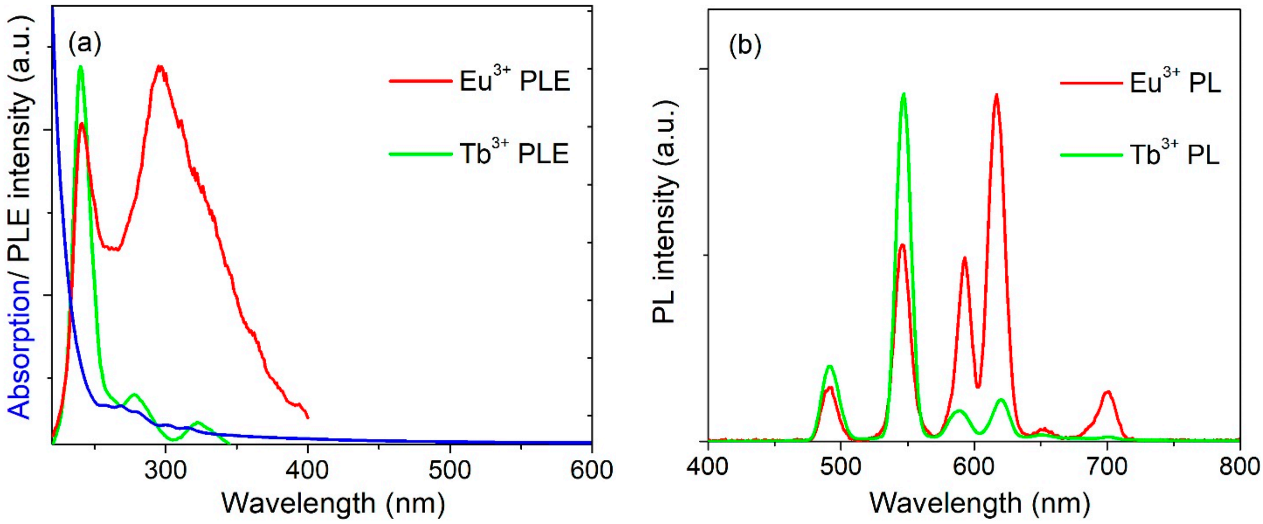

Figure S6. (a) Absorbance/PLE and (b) delayed PL spectra of $Sb^{3+}/Tb^{3+}/Eu^{3+}$-co-doped CNIC NCs with nominal doping concentrations of 0.5%/12.5%/12.5%. For delayed PL measurements, the NCs were excited at 239 nm (corresponding PLE recorded for $^5D_4$–$^7F_5$ Tb^{3+} line at 548 nm) and at 297 nm (corresponding PLE recorded for $^5D_0$–$^7F_2$ Eu^{3+} line at 617 nm).

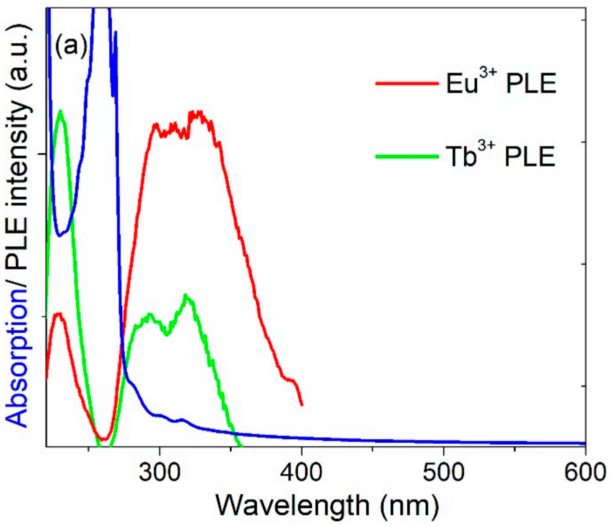

Figure S7. (a) Absorbance/PLE and (b) delayed PL spectra of $Sb^{3+}/Tb^{3+}/Eu^{3+}$-co-doped CNYC NCs with nominal doping concentrations of 0.5%/12.5%/12.5%. For delayed PL measurements, the NCs were excited at 232 nm (corresponding PLE recorded for $^5D_4$–$^7F_5$ Tb^{3+} line at 548 nm) and at 297 nm (corresponding PLE recorded for $^5D_0$–$^7F_2$ Eu^{3+} line at 617 nm).

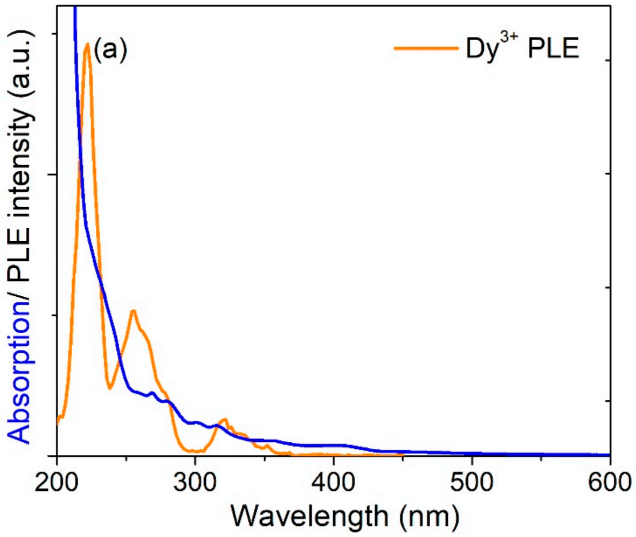

Figure S8. (a) Absorbance/PLE and (b) delayed PL spectra of 12.5% Dy^{3+}-doped CNGC NCs. Dy^{3+} can be excited through the NC host lattice at 220 nm. Emission lines are assigned to the transitions from $^4F_{9/2}$ to $^6H_{15/2}$, $^6H_{13/2}$, $^6H_{11/2}$, and $^6H_{9/2}$, corresponding to wavelengths of 484 nm, 578 nm, 670 nm, and 759 nm, respectively.

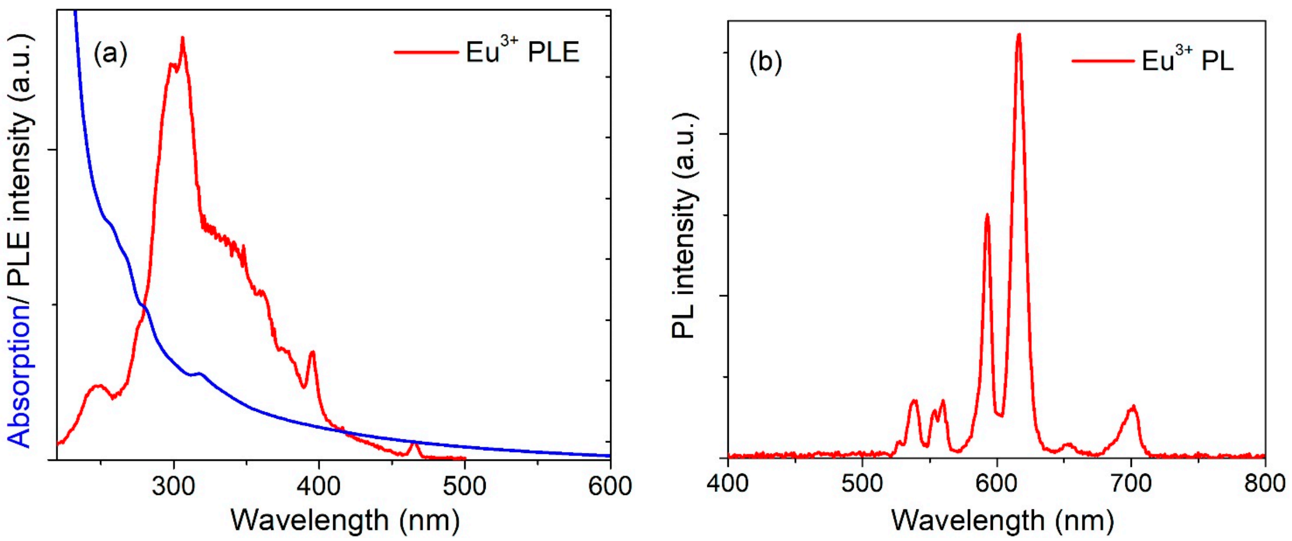

Figure S9. (a) Absorbance/PLE and (b) delayed PL spectra of 12.5% Eu^{3+}-doped CNIC NCs. Eu^{3+} can be excited through the NC host lattice at 300 nm. Additional sharp lines in the PLE spectrum correspond to intrinsic Eu^{3+} excitation (*e.g.*, $^7F_0$ to $^5L_6$ and $^5D_2$ at 394 nm and 464 nm, respectively). Emission lines are assigned to the transitions from $^5D_0$ to $^7F_0$, $^7F_1$, $^7F_2$, $^7F_3$, and $^7F_4$, corresponding to wavelengths of approximately 540-560 nm, 594 nm, 617 nm, 654 nm, and 703 nm, respectively.

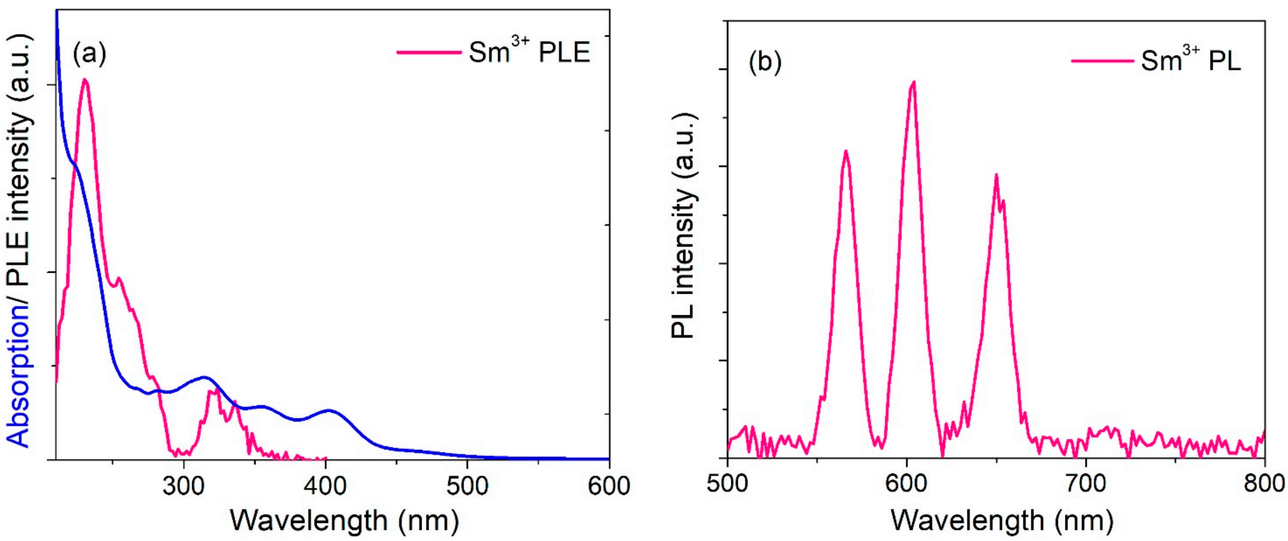

Figure S10. (a) Absorbance/PLE and (b) delayed PL spectra of 12.5% Sm^{3+}-doped CNGC NCs. Sm^{3+} can be excited through the NC host lattice at 230 nm. Emission lines are assigned to the transitions from $^4G_{5/2}$ to $^6H_{5/2}$, $^6H_{7/2}$, and $^6H_{9/2}$, corresponding to wavelengths of 566 nm, 602 nm, and 650 nm, respectively.

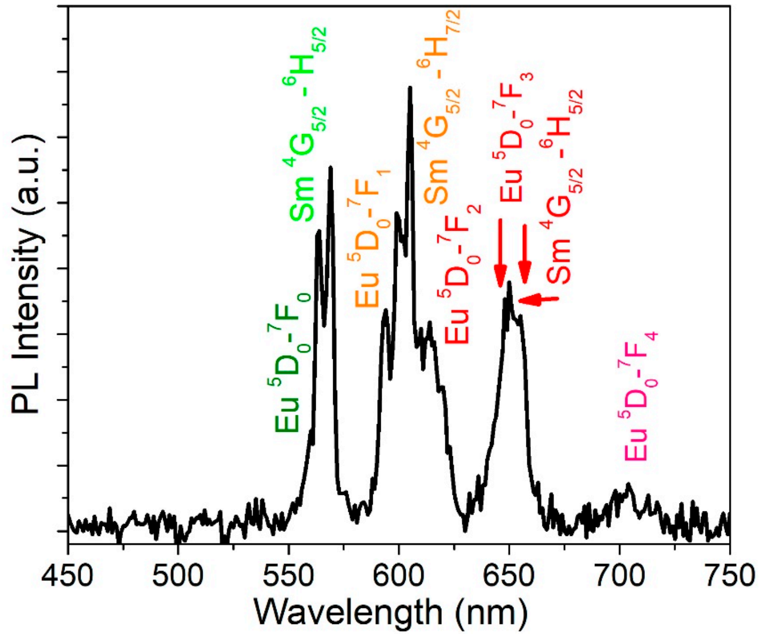

Figure S11. Delayed PL spectra of Sb^{3+}/Sm^{3+}/Eu^{3+}-co-doped CNYC DHP NCs with nominal doping concentrations of 5%/12.5%/12.5%. The NCs were excited at 297 nm, and the PL spectra were recorded with a narrow-slit width (1 nm). The samarium ($^4G_{5/2} \rightarrow {}^6H_{5/2}$, $^6H_{7/2}$, and $^6H_{9/2}$) and europium ($^5D_0 \rightarrow {}^7F_0$, $^7F_1$, $^7F_2$, and $^7F_3$) lines strongly overlap, rendering selective excitation of lanthanide dopants impractical.

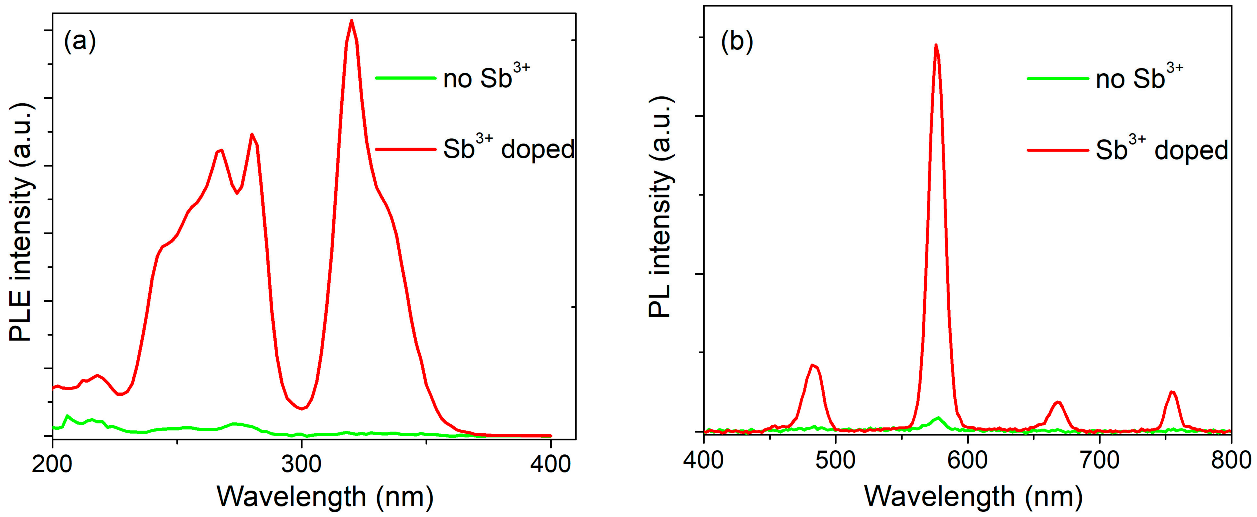

Figure S12. PLE (a) and PL (b) spectra of 12.5% Dy^{3+}-doped, and Sb^{3+}/Dy^{3+}-co-doped with nominal doping concentrations of 5%/12.5% CNGC NCs. The PL spectra were excited at 220 nm, and the PLE intensity was monitored at 576 nm ($^4F_{9/2}$–$^6H_{13/2}$ Dy^{3+} line).

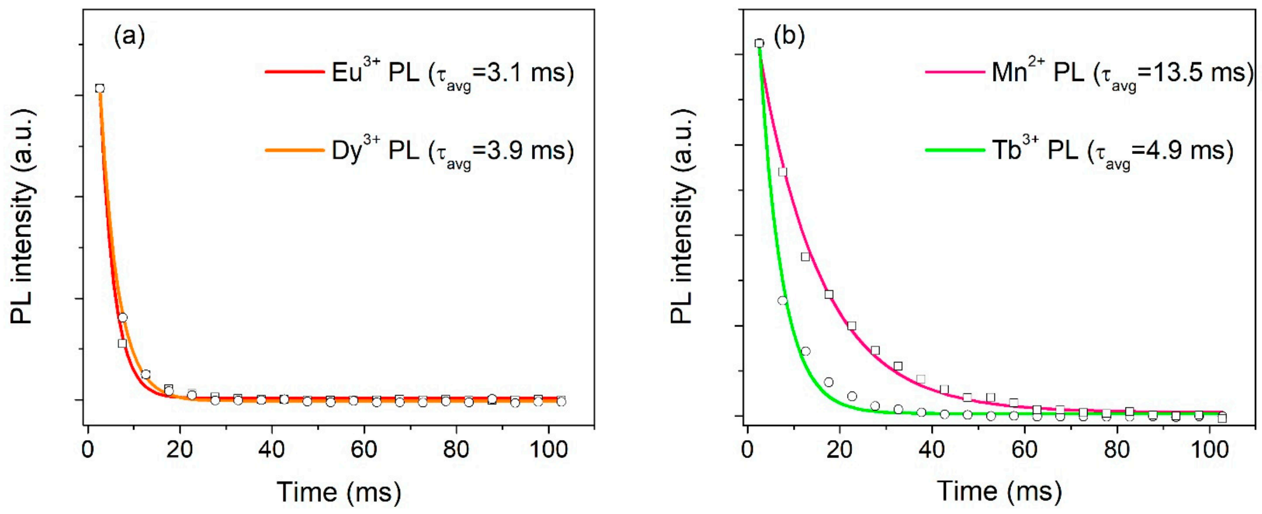

Figure S13. TRPL decay data for (a) $Sb^{3+}/Dy^{3+}/Eu^{3+}$-co-doped CNIC NCs with a nominal doping concentration of 5%/12.5%/12.5% and (b) $Sb^{3+}/Tb^{3+}/Mn^{2+}$-co-doped CNIC NCs with a nominal doping concentration of 5%/16.7%/8.3%. The NCs in (a) were excited at 322 nm, and the PL signal intensities were recorded at 576 nm ($^4F_{9/2} \rightarrow {}^6H_{13/2}$ Dy^{3+} line) and 617 nm ($^5D_0 \rightarrow {}^7F_2$ Eu^{3+} line). The NCs in (b), excited at 322 nm, the PL signal intensities were recorded at 548 nm ($^5D_4 \rightarrow {}^7F_5$ Tb^{3+} line) and 622 nm ($^4T_1 \rightarrow {}^6A_1$ Mn^{2+} d-d transition).

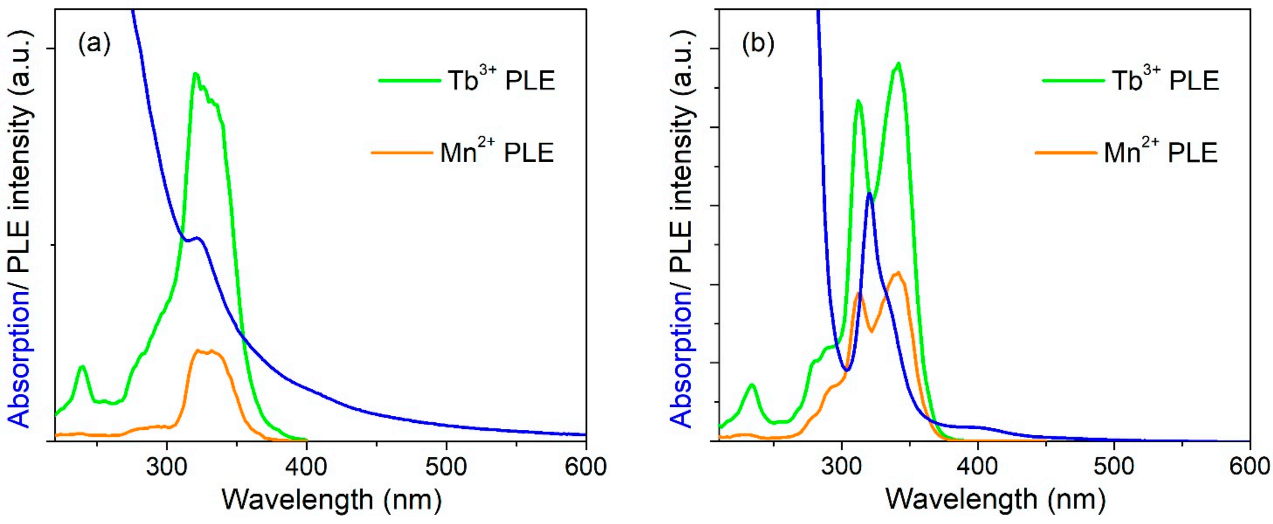

Figure S14. Absorption and PLE spectra of $Sb^{3+}/Tb^{3+}/Mn^{2+}$-co-doped (a) CNIC and (b) CNGC NCs with nominal doping concentrations of 5%/16.7%/8.3%. The NCs were excited at 322 nm, and the PL intensity was monitored at 548 nm ($^5D_4$–$^7F_5$ Tb^{3+} line) and 622 nm ($^4T_1$–$^6A_1$ Mn^{2+} d-d transition).

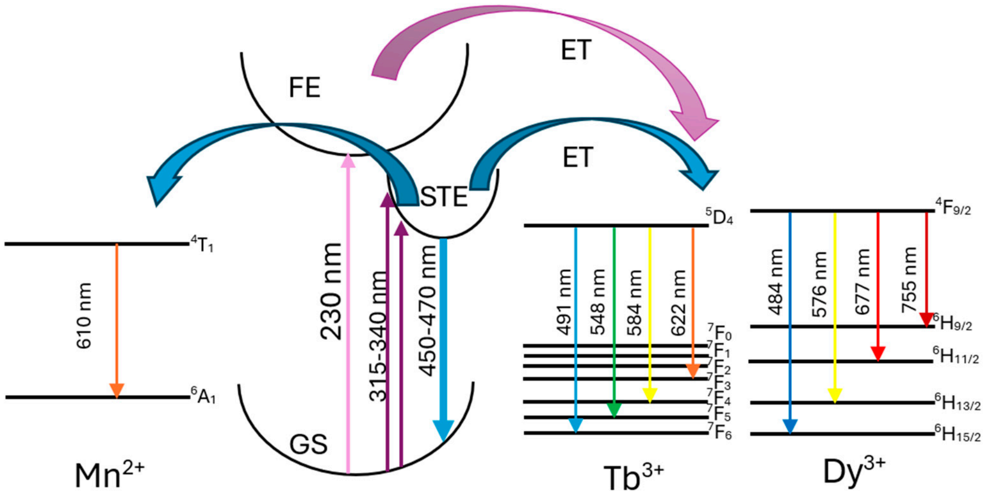

Figure S15. Schematic representation of the excitation and emission pathways for different co-dopants in DHP NCs studied in this work.

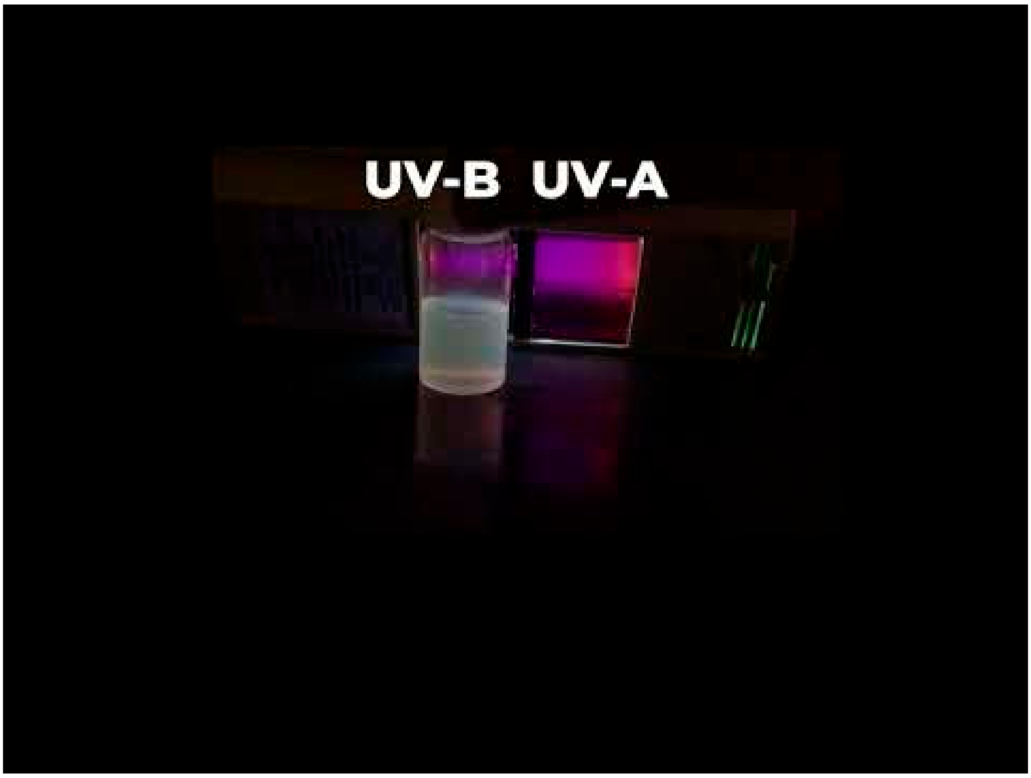

Video S1. Qualitative demonstration of the selective excitation of $Sb^{3+}/Tb^{3+}/Eu^{3+}$-co-doped CNYC NCs with nominal doping concentrations of 5%/12.5%/12.5%. The colloidal NC sample was excited by UV-A/UV-B radiation using UVGL-15 lamp.
